# Supervised physical therapy versus surgery for patients with lumbar spinal stenosis: a propensity score-matched analysis

**DOI:** 10.1186/s12891-022-05632-y

**Published:** 2022-07-11

**Authors:** Masakazu Minetama, Mamoru Kawakami, Masatoshi Teraguchi, Yoshio Enyo, Masafumi Nakagawa, Yoshio Yamamoto, Sachika Matsuo, Tomohiro Nakatani, Nana Sakon, Yukihiro Nakagawa

**Affiliations:** 1 Spine Care Center, Wakayama Medical University Kihoku Hospital, 219 Myoji, Katsuragi-cho, Ito-gun, Wakayama, 649-7113 Japan; 2 Department of Orthopaedic Surgery, Saiseikai Wakayama Hospital, 45 Jyunibancho, Wakayama city, Wakayama, 640-8158 Japan

**Keywords:** Lumbar spinal stenosis, Physical therapy, Exercise therapy, Supervised exercise, Decompression surgery, Lumbar fusion, Propensity score

## Abstract

**Background:**

Previous studies comparing surgical with nonsurgical treatment for lumbar spinal stenosis (LSS) reported that surgery is superior to nonsurgical treatments, but intensive and adequate volume of physical therapy were rarely performed. The purpose of this study was to compare the 1-year follow-up outcomes of patients with LSS treated with supervised physical therapy or surgery using propensity score-matched analysis.

**Methods:**

A total of 224 patients with LSS who received supervised physical therapy (*n* = 38) or surgery (*n* = 186) were included, of which 66 were matched on baseline demographics, radiological findings, and patient-reported outcomes. The physical therapy group received supervised physical therapy twice weekly for 6 weeks. The physical therapy sessions included manual therapy, individually tailored exercises, cycling, and body-weight supported treadmill walking. The surgery group underwent decompression surgery with or without spinal fusion. A propensity score analysis was performed using a one-to-one nearest neighbor approach.

**Results:**

The surgery group showed greater improvements in Zurich claudication questionnaire symptom severity and physical function, SF-36 physical functioning, bodily pain, and mental health, but had more severe stenosis and symptoms and mental health problems than the physical therapy group at baseline (*P* <  0.05). After propensity score matching, there were no significant differences in baseline characteristics, and all clinical outcomes at 1 year, except for a higher percentage of responders achieving minimum clinically important difference in the role-emotional subscale of SF-36 in the surgery group (*P* <  0.05).

**Conclusions:**

When baseline characteristics were considered, supervised physical therapy yielded similar effects to lumbar surgery. These results suggest that supervised physical therapy is preferred over surgery as first-choice treatment, to prevent complications and to minimize health care costs, especially in mild to moderate cases of LSS.

## Background

Lumbar spinal stenosis (LSS) is the most common indication for spinal surgery in older adults [1]. Decompression surgery with or without spinal fusion is the method of choice for LSS, spinal fusion in particular being increasingly performed [2]. Degenerative spondylolisthesis is one of the common causes of LSS and reasons for LSS surgery [1–3]. Nonsurgical treatment is typically recommended before surgery because symptoms or neurological function are unlikely to worsen or deteriorate rapidly [3]. If sufficiently bothersome symptoms persist after nonsurgical treatments, decompression surgery is generally considered.

Various nonsurgical treatments exist, including oral medications, injections, bracing, and physical therapy, consisting of both exercise and manual therapy, and patient education [3]. Exercise therapy is one of the most often recommended nonsurgical treatments for patients with LSS [4]. Manual therapy with supervised exercise improves short-term walking capacity, pain, and symptom severity compared with self-directed or group exercise [5, 6]. Furthermore, supervised physical therapy produces greater improvements in symptom severity and physical function than unsupervised exercise and is associated with a lower likelihood of receiving surgery within 1 year [7].

Compared with nonsurgical treatment, decompression surgery has been reported to produce a greater improvement in disability, but with a higher rate of adverse events [8]. In contrast, in studies of nonsurgical treatment, different modalities, including bracing, physical therapy, and injections were applied in various ways [8]. In the largest clinical trial, the Spine Patient Outcomes Research Trial (SPORT), which included both a randomized and a concurrent observational cohort of patients with LSS, only 37% of patients in the nonsurgical group received physical therapy in the first 6 weeks [9]. A randomized controlled trial (RCT) comparing supervised physical therapy with surgery for LSS showed that physical therapy yielded similar effects to decompression surgery, although about half of the patients assigned to physical therapy crossed over to the surgery group [10]. However, it is unknown how effective supervised physical therapy is compared with decompression surgery with or without fusion for LSS. The purpose of this study was to compare the 1-year follow-up outcomes of LSS patients treated with supervised physical therapy or surgery using a propensity score-matched analysis.

## Methods

### Study design and population

This case-control study was a secondary analysis of an RCT that compared supervised physical therapy with unsupervised exercise for patients with LSS [7]. To assess the outcomes of LSS patients treated with supervised physical therapy or surgery, the original data was compared with the prospectively collected data of patients who underwent surgery for LSS at the same hospital. This study received approval from the institutional review board of Wakayama Medical University (No. 1426, 2728), and was performed in accordance with the Declaration of Helsinki (2013). All patients provided written informed consent before treatment.

Patients treated with supervised physical therapy twice a week for 6 weeks in an RCT were included in the PT group. The physical therapy sessions included manual therapy, individually tailored stretching and strengthening exercises, cycling, and body-weight supported treadmill walking. Patients were received individualized training sessions and treated with same physical therapists during 6 weeks. The selection of the manual therapy and individually tailored exercise was based on the underlying impairments in each patient identified by the treating physical therapist. The manual therapy and individually tailored exercise were aimed to improve mobility and muscle strength of thoracic and lumbar spine and lower extremity to control the posture to minimize symptoms while standing and walking [11]. Each cycling and body-weight supported treadmill walking were performed up to 20 minutes to improve overall fitness and function [11]. In addition to the physical therapy sessions, patients were asked to perform a home-based exercise program consisting of walking and flexion and strengthening exercises during the 6-week intervention [7]. The compliance of a home exercise in flexion and strengthening exercise was 39.1/42 days and 1.9 sessions/day. More information about physical therapy program is available in a previous publication [5]. The surgery group included patients who underwent decompression surgery with or without spinal fusion from September 2014 to May 2018, the same period as the PT group. The patients who had a history of ineffective responses to conservative treatments including pharmacotherapy, treatment with epidural steroid injection or selective nerve root infiltration, physical therapy less than twice a week, or the alternative health care containing massage, thermotherapy and electrotherapy for more than 3 months, and who wished to undergo surgery were enrolled.

The surgery group received postoperative physical therapy during admission. All patients in the PT and surgery groups were allowed to continue with physical therapy once a week or less, which consisted of manual and exercise therapy after the 6-week intervention or discharge.

Inclusion criteria were as follows: (1) age > 50 years, (2) the presence of neurogenic intermittent claudication and pain and/or numbness in the lower extremities with or without low back pain, (3) LSS confirmed by magnetic resonance imaging (MRI), and (4) a history of ineffective responses to pharmacotherapy for more than 3 months. The exclusion criteria were as follows: loss to follow-up at 1 year, receiving surgery or additional surgery within 1-year follow-up, previous spine surgery, degenerative scoliosis, compression fractures at the level being considered for decompression, isthmic spondylolisthesis, severe osteoarthrosis of the knee and/or hip, peripheral artery disorders, cognitive impairment, or a history of psychiatric illness. In the PT group, screening for eligibility was performed by one of three orthopedic spine surgeons in our Spine Care Center. In the surgery group, the eligibility was assessed on medical records. Thirty-eight of 43 patients in PT group (88%) and 186 of 238 patients in the surgery group (78%), who had 1-year follow-up data without receiving surgery within 1 year were included in this study.

### Measurements

The primary outcome was the the Zurich Claudication Questionnaire (ZCQ) [12]. Secondary outcomes included: a numerical rating scale (NRS) of low back pain, leg pain, and leg numbness; the Japanese Orthopaedic Association Back Pain Evaluation Questionnaire (JOABPEQ) [13]; and the Medical Outcomes Study 36-item Short-Form General Health Survey (SF-36) [14] at 1 year. The Hospital Anxiety and Depression Scale (HADS) [15], Pain Catastrophizing Scale (PCS) [16], and Pain Anxiety Symptoms Scale (PASS-20) [17] were used to evaluate psychological status at baseline.

The severity of spinal stenosis was evaluated based on the rootlet/cerebrospinal fluid ratio as seen on T2 axial MRI according to the Schizas classification [18]. Grades A1–A4 indicate no or minor stenosis; grade B, moderate stenosis; grade C, severe stenosis; and grade D, extreme stenosis. Two orthopedic spine surgeons who were certified as specialists by the Japanese Orthopedic Association and the Japanese Society for Spine Surgery and Related Research determined the grade of dura mater compression and reached a consensus for all patients. The intra- and interobserver kappa values were 0.77 and 0.68, respectively. The presence and percent of slippage (% slip) were evaluated using lumbar flexion–extension radiographs of the patient in the standing position. The intra- and interclass correlation coefficient values for slippage were 0.96 and 0.83, respectively. The intra- and interobserver kappa values for the presence of slippage were 0.89 and 0.80, respectively.

### Statistical analysis

To create comparable groups, propensity score matching (PSM) was used, including age, sex, body mass index, duration of symptoms, number of stenoses, % slip, presence of slippage, ZCQ symptom severity and physical function, NRS of low back pain, leg pain, and leg numbness, and SF-36 physical functioning, bodily pain, and mental health at baseline [19]. Binary logistic regression was used for estimating the propensity score using independent variables described above. A propensity score analysis was performed using a one-to-one nearest neighbor approach with a caliper width of 0.2 [20]. Before and after PSM, clinical outcomes at baseline and 1 year were compared between the groups using the Mann–Whitney U test for nonparametric variables, Student’s t test for parametric variables and the χ^2^ test. Post-hoc power analyses for the primary outcome of ZCQ as continuous variables were performed with a medium effect size (Cohen’s *d* = 0.5) to examine the power before and after PSM [21]. Responder analyses showing the percentage of patients achieving minimum clinically important difference (MCID) values were also performed using the χ^2^ test. The MCID for the ZCQ, 0.5 points for symptom severity and physical function subscales, and 2.5 points or less for the satisfaction subscale were used based on the previously published values that indicate a successful outcome for surgery [22]. The MCID for each domain of JOABPEQ was defined as 20 points [23]. The MCID for other outcomes was defined as at least a 30% improvement between their baseline and follow-up scores [24]. All statistical tests were two-tailed, and the significance level was fixed at 0.05 throughout. All computations were performed using JMP Pro (version 14; SAS Institute, Cary, NC, USA).

## Results

Thirty-eight patients (17 male and 21 female, average age 72.5 years) in the PT group and 186 patients (92 male and 94 female, average age 70.9 years) in the surgery group were included in this study (Table 1). The details of surgical treatments and adverse events are shown in Table 2.

**Table 1:** Comparison of demographic data between the physical therapy and surgery groups at the baseline

	Before matching			After matching		
	Physical therapy (***n*** = 38)	Surgery (***n*** = 186)	***P***	Physical therapy (***n*** = 33)	Surgery (***n*** = 33)	***P***
**Age (years)**	72.5 ± 7.0	70.9 ± 8.4	0.28*	72.1 ± 7.3	71.1 ± 7.9	0.80^‡^
**Sex**	Female: 21	Female: 94	0.60^†^	Female: 19	Female: 19	1.00^†^
	Male: 17	Male: 92		Male: 14	Male: 14	
**Body mass index (kg/m**^**2**^**)**	23.4 ± 2.8	23.9 ± 3.3	0.38^‡^	23.5 ± 2.9	23.8 ± 3.4	0.73*
**Duration of symptoms (months)**	31.2 ± 29.7	25.2 ± 27.7	0.10^‡^	26.7 ± 26.0	25.8 ± 28.0	0.61^‡^
**Comorbidities, n (%)**						
**Hypertension**	25 (66)	121 (65)	0.93^‡^	22 (67)	17 (52)	0.21^†^
**Diabetes**	10 (26.3)	45 (24.2)	0.78^‡^	9 (27)	7 (21)	0.57^†^
**Heart disease**	8 (21)	27 (15)	0.31^‡^	7 (21)	4 (12)	0.32^†^
**Pulmonary disease**	4 (11)	12 (7)	0.37^‡^	4 (12)	1 (3)	0.16^†^
**Smoking, n (%)**			0.25^‡^			0.42^†^
**Never**	25 (66)	130 (70)		23 (70)	26 (79)	
**ex-smoker**	11 (29)	35 (19)		8 (24)	4 (12)	
**current smoker**	2 (5)	21 (11)		2 (6)	3 (9)	
**Severity of stenosis at most stenotic level**			< 0.01^†^			< 0.17^†^
**Minor (Grade A)**	7 (18)	10 (5)		6 (18)	1 (3)	
**Moderate (Grade B)**	15 (40)	50 (27)		12 (36)	12 (36)	
**Severe (Grade C)**	16 (42)	121 (65)		15 (46)	19 (58)	
**Extreme (Grade D)**	0 (0)	5 (3)		0 (0)	1 (3)	
**Total number of severe stenoses (more than grade C)**	0.6 ± 0.8	1.0 ± 0.9	< 0.01^‡^	0.7 ± 0.9	0.8 ± 0.7	0.42^‡^
**Spondylolisthesis, n (%)**	25 (66)	108 (58)	0.38^†^	21 (64)	24 (73)	0.43^†^
**% slippage (mm)**	7.2 ± 6.8	8.9 ± 9.5	0.50^‡^	7.5 ± 7.2	8.4 ± 7.6	0.75^‡^
Severe LSS symptoms*	6 (16)	62 (33)	0.03^†^	5 (15)	7 (21)	0.52^†^
**Zurich claudication questionnaire**						
**Symptom severity**	3.2 ± 0.5	3.5 ± 0.6	0.02^‡^	3.3 ± 0.5	3.3 ± 0.6	0.52^‡^
**Physical function**	2.4 ± 0.6	2.7 ± 0.6	0.01^‡^	2.4 ± 0.6	2.4 ± 3.1	0.97*
**Numerical rating scale**						
**Back pain**	5.3 ± 2.5	5.8 ± 2.7	0.21^‡^	5.1 ± 2.5	5.2 ± 2.5	0.77*
**Leg pain**	6.4 ± 2.2	6.8 ± 2.6	0.17^‡^	6.4 ± 2.2	6.2 ± 2.7	0.98^‡^
**Leg numbness**	5.7 ± 2.7	5.9 ± 3.0	0.50^‡^	5.9 ± 2.7	6.4 ± 2.7	0.36^‡^
**Japanese orthopedic association back pain evaluation questionnaire**						
**Pain-related disorders**	56.0 ± 30.2	44.2 ± 33.5	0.04^‡^	57.7 ± 30.8	53.3 ± 31.6	0.59^‡^
**Lumbar dysfunction**	63.1 ± 27.7	57.5 ± 28.3	0.27^‡^	62.9 ± 28.8	65.9 ± 23.0	0.81^‡^
**Gait disturbance**	40.7 ± 27.2	31.8 ± 24.8	0.06^‡^	39.5 ± 27.3	41.3 ± 24.9	0.83^‡^
**Social life dysfunction**	44.2 ± 18.1	36.8 ± 20.5	0.03^‡^	44.4 ± 19.1	46.0 ± 17.6	0.89^‡^
**Psychological disorders**	50.9 ± 14.2	43.8 ± 17.5	0.01^‡^	50.3 ± 14.4	48.8 ± 13.8	0.62^‡^
**SF-36**						
**Physical functioning**	60.5 ± 20.3	45.6 ± 22.7	< 0.01^‡^	57.9 ± 20.2	59.1 ± 20.6	0.81*
**Bodily pain**	38.3 ± 14.3	31.1 ± 15.5	< 0.01^‡^	38.8 ± 15.0	39.2 ± 17.5	0.83^‡^
**Role physical**	53.5 ± 25.5	40.3 ± 25.8	< 0.01^‡^	55.5 ± 26.4	50.8 ± 24.8	0.46*
**Role emotional**	61.8 ± 27.5	46.5 ± 30.1	< 0.01^‡^	63.9 ± 28.3	57.3 ± 23.9	0.16^‡^
**Mental health**	62.6 ± 21.6	57.1 ± 20.6	< 0.05^‡^	60.5 ± 21.8	59.4 ± 21.1	0.62^‡^
**Social functioning**	70.7 ± 26.8	59.7 ± 27.7	0.02^‡^	70.1 ± 27.6	69.7 ± 28.1	0.98^‡^
**Vitality**	53.5 ± 19.2	46.7 ± 22.2	0.07^‡^	52.1 ± 20.1	51.9 ± 17.8	0.98^‡^
** General health**	49.3 ± 14.8	49.1 ± 16.7	0.86^‡^	48.8 ± 14.0	50.5 ± 14.1	0.61*
**Hospital anxiety and depression scale**						
**Depression**	5.2 ± 3.1	5.9 ± 3.9	0.46^‡^	5.6 ± 3.1	5.3 ± 2.8	0.72*
**Anxiety**	4.2 ± 2.6	5.7 ± 3.9	0.03^‡^	4.5 ± 2.6	4.4 ± 3.1	0.95^‡^
**Pain catastrophizing scale**	28.0 ± 10.4	31.9 ± 11.0	0.03^‡^	29.4 ± 10.1	30.9 ± 9.7	0.55*
**Pain anxiety symptoms scale-20**	37.1 ± 17.2	43.0 ± 16.8	> 0.05*	39.7 ± 16.2	44.8 ± 15.0	0.20*

Values are mean ± *SD*, *Student’s *t* test, ^†^χ^2^ test, ^‡^Mann–Whitney *U* test.

^§^ Patients with Zurich Claudication Questionnaire symptom severity score exceeding the 75th percentile (≥ 3.8 points) at baseline were defined as severe lumbar spinal stenosis symptoms.

SF-36, Medical Outcomes Study 36-Item Short-Form General Health Survey

**Table 2:** Surgical treatments and adverse events

	Before matching (***n*** = 186)	After matching (***n*** = 33)	***P***
**Decompression only, n (%)**	91 (49)	18 (55)	0.55*
**Decompression with fusion, n (%)**	95 (51)	15 (45)	0.55*
**Decompression with instrumented fusion, n (%)**	52 (27)	7 (21)	0.42*
**Total number of decompression levels**	2.7 ± 1.2	2.6 ± 1.1	0.78^†^
**Total number of fusion levels**	2.4 ± 1.9	2.5 ± 1.4	0.75^†^
**Total number of instrumented fusion levels**	1.2 ± 0.5	1.1 ± 0.4	0.43^†^
**Blood loss (ml)**	589.3 ± 663.5	491.5 ± 504.5	0.79^†^
**Blood replacement, n (%)**	49 (26)	7 (21)	0.53*
**Adverse events, n (%)**			
** Dural tear or spinal fluid leak**	22 (12)	2 (6)	0.54^‡^
** Hematoma required reoperation**	6 (3)	1 (3)	1.00^‡^
** Implant problems required reoperation**	2 (1)	2 (6)	0.11^‡^
** Nerve root damage**	13 (7)	4 (12)	0.30^‡^
** Infection**	5 (3)	1 (3)	1.00^‡^
** Deep vein thrombosis**	1 (1)	1 (3)	1.00^‡^
** Heart failure**	3 (2)	0 (0)	1.00^‡^
** Pneumonia**	2 (1)	0 (0)	1.00^‡^

Values are mean ± SD, *χ^2^ test, ^†^Mann–Whitney *U* test, ^‡^Fisher’s exact test

### Before PSM

At baseline before PSM, the surgery group had more severe stenosis at the most stenotic level, a higher number of severe stenoses (≥ Grade C), and worse scores on ZCQ symptom severity and physical function, JOABPEQ pain-related disorders, social life dysfunction, and psychological disorders, all domains of SF-36 except for vitality and general health, HADS anxiety, and PCS than the PT group (Tables 1). The proportion of patients with severe LSS symptoms, which indicate ZCQ symptom severity score exceeding the 75th percentile (≥3.8 points) [25] at baseline was higher in the surgery group than in the PT group (33% vs 16%, respectively, *P* = 0.03) (Table 1). Analysis of the mean changes after 1 year before PSM showed that the surgery group underwent significant improvements compared with the PT group for ZCQ symptom severity and physical function, NRS of low back pain, JOABPEQ pain-related disorders, gait disturbance, and psychological disorders, and SF-36 physical functioning and bodily pain (all *P* <  0.05) (Table 3). The responder analyses before PSM showed that greater percentages of patients in the surgery group than in the PT group achieved MCID values in ZCQ physical function, NRS of low back pain, JOABPEQ pain-related disorders, gait disturbance, and psychological disorders, and SF-36 physical functioning, role-physical, role-emotional, and social functioning subscales (all *P* <  0.05) (Table 4). However, the ZCQ satisfaction subscale showed that a greater percentage of patients in the PT group were satisfied with their treatment compared with the surgery group (94.7% vs 76.9%, respectively, *P* <  0.05).

**Table 3:** Comparison of clinical outcomes between the physical therapy and surgery groups at 1 year

	Before matching			After matching		
	Mean change at 1 year		Difference between groups	Mean change at 1 year		Difference between groups
	Physical therapy (***n*** = 38) (95% CI)	Surgery (***n*** = 186) (95% CI)	Physical therapy minus Surgery (95% CI)	Physical therapy (***n*** = 33) (95% CI)	Surgery (***n*** = 33) (95% CI)	Physical therapy minus Surgery (95% CI)
**Zurich claudication questionnaire**						
**Symptom severity**	−0.6 (−0.8 to −0.5)	−0.9 (−1.0 to − 0.8)	0.3 (0.03 to 0.5)*	− 0.7 (− 0.9 to − 0.5)	−0.8 (− 1.1 to − 0.4)	0.1 (− 0.3 to 0.5)
**Physical function**	−0.5 (− 0.7 to − 0.3)	−0.8 (− 0.9 to − 0.7)	0.3 (0.1 to 0.6)*	−0.5 (− 0.7 to − 0.2)	−0.6 (− 0.8 to − 0.3)	0.1 (− 0.2 to 0.5)
**Numerical rating scale**						
**Back pain**	− 0.7 (− 1.8 to 0.4)	− 2.1 (− 2.6 to − 1.6)	1.4 (0.2 to 2.6)*	−0.7 (− 1.9 to 0.5)	− 1.5 (− 2.8 to − 0.2)	0.8 (− 0.9 to 2.5)
**Leg pain**	−2.1 (− 3.1 to − 1.0)	−2.9 (− 3.4 to − 2.4)	0.9 (− 0.4 to 2.1)	−2.1 (− 3.3 to − 0.9)	−2.2 (− 3.4 to − 1.0)	0.1 (− 1.6 to 1.8)
**Leg numbness**	– 1.4 (− 2.8 to − 0.1)	– 2.0 (− 2.5 to − 1.5)	0.6 (− 0.8 to 1.9)	− 1.6 (− 3.0 to − 0.2)	−2.0 (− 3.0 to − 1.1)	0.4 (− 1.3 to 2.1)
**Japanese orthopedic association back pain evaluation questionnaire**						
**Pain-related disorders**	10.2 (−1.0 to 21.4)	30.2 (24.5 to 35.8)	−20.0 (− 33.4 to − 6.6)*	9.5 (− 2.9 to 22.0)	20.1 (5.7 to 34.4)	− 10.5 (− 29.2 to 8.2)
**Lumbar dysfunction**	8.8 (1.9 to 15.7)	10.9 (6.4 to 15.4)	− 2.0 (− 10.2 to 6.1)	8.4 (0.8 to 15.9)	0.3 (− 9.4 to 10.0)	8.0 (− 4.0 to 20.1)
**Gait disturbance**	15.3 (5.8 to 24.9)	32.3 (27.7 to 36.8)	−16.9 (− 27.9 to − 5.9)*	16.2 (5.3 to 27.0)	21.7 (9.4 to 34.0)	−5.6 (− 21.7 to 10.5)
**Social life dysfunction**	13.6 (6.3 to 20.8)	21.9 (18.2 to 25.6)	−8.3 (− 17.0 to 0.4)	13.8 (5.7 to 22.0)	13.1 (3.9 to 22.3)	0.8 (− 11.3 to 12.8)
**Psychological disorders**	5.4 (1.1 to 9.7)	11.9 (9.0 to 14.8)	−6.5 (−11.7 to − 1.4)*	5.7 (1.1 to 10.4)	4.4 (− 3.5 to 12.2)	1.3 (− 7.7 to 10.3)
**SF-36**						
**Physical functioning**	4.6 (−1.0 to 10.2)	19.4 (15.9 to 22.8)	−14.8 (− 21.3 to −8.3)*	6.1 (0.2 to 11.9)	9.4 (−0.7 to 19.5)	−3.4 (− 14.9 to 8.2)
**Bodily pain**	17.0 (10.5 to 23.5)	25.6 (22.0 to 29.2)	−8.6 (− 17.1 to − 0.2)*	16.8 (9.4 to 24.2)	19.3 (11.8 to 26.8)	−2.5 (− 12.8 to 7.8)
**Role physical**	10.5 (1.7 to 19.4)	18.5 (13.9 to 23.1)	−8.0 (− 18.9 to 2.9)	8.9 (− 0.4 to 18.2)	7.9 (− 1.9 to 17.8)	1.0 (− 12.3 to 14.2)
**Role emotional**	6.4 (− 4.1 to 16.8)	15.9 (10.8 to 21.0)	−9.6 (− 21.7 to 2.6)	3.8 (− 7.8 to 15.4)	6.1 (− 4.5 to 16.6)	−2.3 (− 17.6 to 13.1)
**Mental health**	4.9 (− 3.8 to 13.5)	12.5 (9.3 to 15.6)	−7.6 (− 15.5 to 0.3)	7.0 (− 2.3 to 16.2)	8.6 (− 0.4 to 17.7)	−1.7 (− 14.3 to 11.0)
**Social functioning**	5.6 (− 2.8 to 14.0)	13.0 (8.3 to 17.8)	−7.4 (− 18.5 to 3.7)	6.4 (− 2.4 to 15.2)	2.3 (− 8.0 to 12.5)	4.2 (− 9.1 to 17.4)
**Vitality**	5.4 (− 1.2 to 12.0)	13.2 (9.8 to 16.6)	−7.8 (− 15.8 to 0.2)	6.1 (− 1.1 to 13.2)	7.3 (0.4 to14.3)	−1.3 (− 11.1 to 8.5)
**General health**	7.4 (2.1 to 12.7)	6.3 (3.7 to 9.0)	1.1 (− 5.3 to 7.4)	7.2 (1.3 to 13.2)	2.4 (− 4.1 to 8.9)	4.8 (− 3.8 to 13.5)

Values represent mean change from baseline.

* *P* < 0.05

*CI* Confidence interval; *SF*-36 Medical Outcomes Study 36-Item Short-Form General Health Survey.

**Table 4:** Responder analysis (patients achieving MCID)

	Before matching			After matching		
	Patients achieving MCID at 1 year (%)		Difference between groups	Patients achieving MCID at 1 year (%)		Difference between groups
	**Physical therapy** **(*n*** **= 38)** **(95% CI)**	**Surgery** **(*n*** **= 186)** **(95% CI)**	**Physical therapy** **minus Surgery** **(95% CI)**	**Physical therapy** **(*n*** **= 33)** **(95% CI)**	**Surgery** **(*n*** **= 33)** **(95% CI)**	**Physical therapy** **minus Surgery** **(95% CI)**
**Zurich claudication questionnaire**						
**Symptom severity**	57.9	66.1	−8.2 (− 25.2 to 8.3)	57.6	63.6	−6.1 (− 28.6 to 17.2)
**Physical function**	42.1	67.2	−25.1 (− 41.3 to −7.8)*	42.4	60.6	−18.2 (− 40.2 to 5.9)
**Satisfaction**	94.7	76.9	17.9 (5.7 to 26.1)*	93.9	78.8	15.2 (−2.4 to 31.0)
**Numerical rating scale**						
**Back pain**	29.0	52.7	−23.7 (−38.6 to −6.8)*	30.3	45.5	−15.1 (− 36.8 to 8.3)
**Leg pain**	52.6	58.1	−5.4 (−22.5 to 11.5)	51.5	54.6	−3.0 (−26.2 to 20.5)
**Leg numbness**	39.5	50.5	−11.1 (−27.3 to 6.2)	39.4	45.5	−6.0 (−28.9 to 17.4)
**Japanese orthopedic association back pain evaluation questionnaire**						
**Pain-related disorders**	47.1	70.2	−23.2 (−40.5 to −5.8)*	44.8	69.0	−24.1 (−46.6 to 1.5)
**Lumbar dysfunction**	44.1	46.9	−2.7 (− 20.3 to 15.3)	44.8	25.8	19.0 (−5.3 to 41.1)
**Gait disturbance**	45.7	68.9	−23.2 (−40.1 to −5.3)*	45.2	53.1	−8.0 (−31.3 to 16.4)
**Social life dysfunction**	34.2	49.7	−15.5 (− 31.1 to 1.7)	36.4	39.4	−2.6 (− 25.7 to 20.0)
**Psychological disorders**	13.2	32.8	−19.6 (−30.9 to −5.0)*	12.1	21.2	−9.1 (−26.7 to 9.5)
**SF-36**						
**Physical functioning**	29.0	58.6	−29.7 (−44.4 to −12.7)*	33.3	42.4	−9.1 (−31.2 to 14.1)
**Bodily pain**	55.3	69.9	−14.6 (−31.4 to 2.1)	51.5	60.6	−9.1 (−31.8 to 14.6)
**Role physical**	36.8	56.5	−19.6 (−35.5 to −2.3)*	34.4	48.4	−14.0 (−36.6 to 10.2)
**Role emotional**	23.7	49.5	−25.8 (−39.7 to −9.3)*	19.4	44.8	−25.5 (−46.3 to −1.6)*
**Mental health**	31.6	41.4	−9.8 (−25.1 to 7.1)	33.3	36.4	−3.0 (−25.3 to 19.6)
**Social functioning**	23.7	45.7	−22.0 (−35.9 to −5.6)*	29.6	33.3	−3.7 (− 26.9 to 20.2)
**Vitality**	34.2	44.1	−9.9 (−25.5 to 7.2)	36.4	30.3	6.1 (−16.5 to 27.9)
**General health**	23.7	34.4	−10.7 (−24.6 to 5.5)	21.2	21.2	0 (−19.7 to 19.7)

* *P* < 0.05

*MCID* Minimal clinically important difference; *CI* Confidence interval; *SF*-36 Medical Outcomes Study 36-Item Short-Form General Health Survey

### After PSM

Thirty-three pairs were selected by PSM with the propensity score AUC value of 0.80. After PSM, there were no significant differences in all clinical outcomes at baseline and 1 year, except for the percentage of responders achieving MCID value in SF-36 role-emotional subscale (PT group; 19.4% vs surgery group; 44.8%, *P* <  0.05) (Tables 1, 3, 4). Types of surgical procedures and proportion of adverse events did not differ before and after PSM among the surgery group (Table 2). Post hoc power analyses detected that the current sample size had a power of 0.88 for before PSM and 0.64 for after PSM.

## Discussion

Our study showed that patients who underwent decompression surgery with or without fusion showed greater improvements in clinical outcomes, but had more severe stenosis and LSS symptoms, and mental health problems than patients who received supervised physical therapy at baseline. However, when the proportions of patients with severe stenosis and LSS symptoms were equivalent after matching for baseline characteristics, supervised physical therapy yielded similar improvements to decompression surgery among patients with LSS.

A previous study comparing surgery with nonsurgical treatment for LSS using PSM showed that patients who underwent nonsurgical treatment had lower quality of life at the 1-year follow-up as well as a lower chance of reaching an MCID in ZCQ symptom severity and function than patients who underwent surgery [19]. However, in that study, nonsurgical treatment was not uniform and it is unclear how many patients received physical therapy and which type and amount of physical therapy was performed. Six weeks of supervised physical therapy twice a week for patients with LSS has been reported to produce greater improvements in symptom severity, physical function, back and leg pain, and gait disturbance than with once a week and/or home exercise alone [26]. Several studies comparing surgical with nonsurgical treatment for LSS reported that surgery is superior to nonsurgical treatments, but intensive and adequate volume of physical therapy were not performed for all participants [27, 28]. On the other hand, an RCT comparing 6 weeks of supervised physical therapy twice a week with surgery for LSS showed that physical therapy yielded similar effects to decompression surgery without fusion [10]. Therefore, clinical outcomes of nonsurgical treatments might differ depending on whether an adequate volume of supervised physical therapy was performed.

At baseline, patients who underwent surgery had more severe stenosis and LSS symptoms than patients who received supervised physical therapy. Decompression surgery produced better clinical outcomes compared with supervised physical therapy at 1 year. The same trends were observed in the SPORT study, which compared surgery with nonsurgical treatments for LSS [28]. In that study, patients who underwent surgery had more severe stenosis, pain, self-reported disability, and psychological distress, and had a lower level of function than patients who did not receive surgery. Moreover, intent-to-treat for the randomized cohort and adjusted analyses according to treatment received for the randomized and observational cohorts combined, patients who underwent surgery showed greater improvements in SF-36 physical function and bodily pain, and Oswestry disability index than patients treated with nonsurgical treatments through 4 years [28]. Given that decompression surgery resulted in similar clinical outcomes to supervised physical therapy when baseline severity of symptoms was matched, patients with severe symptoms might be more likely to obtain therapeutic benefit from surgery than moderate cases. On the other hand, our study showed that the satisfaction rate was higher in the PT group than in the surgery group. Moreover, several adverse events were observed in the surgery group. When baseline characteristics were matched, surgery was not superior over physical therapy, except the role-emotional subscale of SF-36. Our findings indicate that supervised physical therapy could be preferred over surgery as first-choice treatment, to prevent complications and to minimize health care costs, especially in mild to moderate cases of LSS.

The natural history of LSS with moderate symptom levels has shown that about one-third of patients reported improvement of back and leg pain and walking ability. Symptoms of half of the patients were unchanged while worsening was noted in 10–13% for pain levels and 22% for walking ability [29]. Neither degenerative spondylolisthesis, degenerative scoliosis, nor the number of stenotic levels influenced the natural history, although patients with dural sac area < 0.5 cm^2^ did not show improvements to the same extent as those seen in patients with dural sac area ≥ 0.5 cm^2^ [29]. In the present study, patients who received supervised physical therapy had less severe stenosis than patients who underwent surgery. The lower prevalence rate of severe stenosis might affect the results in that similar effects were observed between surgery and supervised physical therapy when radiological findings and LSS symptoms were matched.

Although the duration of preoperative nonsurgical treatment has been reported to be not associated with the ultimate outcome of decompression surgery [30], other studies showed that preoperative longer duration of symptoms, lower function, and narrower dural sac area predict inferior outcomes in terms of pain, numbness, function, and quality of life [31, 32]. On the other hand, early surgical decompression provided better recovery rate and neurological improvement for foot drop in lumbar degenerative diseases than late surgical decompression [33]. Therefore, patients with severe spinal canal stenosis and/or lower extremity paralysis might be better to consider the early decompression surgery. Future studies should confirm whether the early surgery should be performed before or after intensive physical therapy.

This study has some limitations. The 1-year follow-up period without paralysis assessment was insufficient for evaluating the effectiveness of surgical and nonsurgical treatment for LSS. Previous RCTs that have evaluated physical therapy for LSS showed that clinical outcomes remain stable up to 2 years after a 6-week supervised physical therapy intervention [10, 34]. On the other hand, the studies comparing surgery with nonsurgical treatment for LSS showed that the relative benefit of surgery diminished with time after 2 to 4 years [27, 35]. Future studies with long-term follow-up including paralysis assessment are needed. The sample size in the PT group was small, and the surgery group had five times as many patients as the PT group. Caution should be taken in interpreting the results, because the powers were 0.88 for before PSM and 0.64 for after PSM (Cohen’s *d* = 0.5). In the PT group, one patient who underwent decompression surgery within the 1-year follow-up period, two patients who dropped out, and two patients who did not answer the self-reported questionnaire at 1 year were excluded [7]. Therefore, there is selection bias due to a lack of intention-to-treat analysis. Unlike randomization, propensity score methods only ensure balance in measured, not unmeasured confounders. Although 15 variables were used for PSM based on previous study [19], the influence of unmeasured confounder, such as patient preference cannot be ruled out. The results should be interpreted with caution. In the present study, all patients in the surgery group were received postoperative physical therapy during admission, however, the number and duration of continued physical therapy after discharge were not assessed. The responder analyses after PSM showed that the surgery group had a higher percentage of responders in the SF-36 role-emotional subscale than the PT group, although the mean changes after 1 year on SF-36 role-emotional subscale did not differ between the groups. It is possible that a lower baseline score, but not the significant difference in the surgery group, affected the higher responder rate at 1 year (57.3 points vs 63.9 points, *P* = 0.16).

## Conclusions

Patients who underwent decompression surgery with or without fusion showed greater improvements in clinical outcomes but had more severe stenosis and LSS symptoms than the patients who received supervised physical therapy at baseline. When baseline characteristics were considered, supervised physical therapy yielded similar effects to lumbar surgery among patients with LSS. These results suggest that supervised physical therapy is preferred over surgery as first-choice treatment, to prevent complications and to minimize health care costs, especially in mild to moderate cases of LSS.

## Data Availability

The datasets used and/or analysed during the current study are available from the corresponding author on reasonable request.
